# Frailty and falls among adult patients undergoing chronic hemodialysis: a prospective cohort study

**DOI:** 10.1186/1471-2369-14-224

**Published:** 2013-10-16

**Authors:** Mara A McAdams-DeMarco, Sunitha Suresh, Andrew Law, Megan L Salter, Luis F Gimenez, Bernard G Jaar, Jeremy D Walston, Dorry L Segev

**Affiliations:** 1 Department of Surgery, Johns Hopkins University School of Medicine, Baltimore, MD, USA; 2 Department of Epidemiology, Johns Hopkins School of Public Health, Baltimore, MD, USA; 3 Johns Hopkins School of Medicine, Baltimore, MD, USA; 4 Nephrology Center of Maryland, Baltimore, MD, USA; 5 Department of Medicine, Division of Nephrology, Johns Hopkins University School of Medicine, Baltimore, MD, USA; 6 Dialysis Program, Good Samaritan Hospital, Baltimore, MD, USA; 7 Division of Geriatric Medicine and Gerontology, Johns Hopkins University School of Medicine, Baltimore, MD, USA; 8 Clinical Research, Transplant Surgery, Johns Hopkins Medical Institutions, 720 Rutland Ave, Ross 771B, Baltimore, MD 21205, USA

**Keywords:** Hemodialysis, Falls, Frailty

## Abstract

**Background:**

Patients undergoing hemodialysis are at high risk of falls, with subsequent complications including fractures, loss of independence, hospitalization, and institutionalization. Factors associated with falls are poorly understood in this population. We hypothesized that insights derived from studies of the elderly might apply to adults of all ages undergoing hemodialysis; we focused on frailty, a phenotype of physiological decline strongly associated with falls in the elderly.

**Methods:**

In this prospective, longitudinal study of 95 patients undergoing hemodialysis (1/2009-3/2010), the association of frailty with future falls was explored using adjusted Poisson regression. Frailty was classified using the criteria established by Fried et al., as a combination of five components: shrinking, weakness, exhaustion, low activity, and slowed walking speed.

**Results:**

Over a median 6.7-month period of longitudinal follow-up, 28.3% of study participants (25.9% of those under 65, 29.3% of those 65 and older) experienced a fall. After adjusting for age, sex, race, comorbidity, disability, number of medications, marital status, and education, frailty independently predicted a 3.09-fold (95% CI: 1.38-6.90, *P*=0.006) higher number of falls. This relationship between frailty and falls did not differ for younger and older adults (*P*=0.57).

**Conclusions:**

Frailty, a validated construct in the elderly, was a strong and independent predictor of falls in adults undergoing hemodialysis, regardless of age. Our results may aid in identifying frail hemodialysis patients who could be targeted for multidimensional fall prevention strategies.

## Background

Patients undergoing chronic hemodialysis are at high risk of falls: 13-25% experience a fall after hemodialysis initiation [1,2]. This population is also at high-risk for severe complications after a fall including fractures, loss of independence, hospitalization, institutionalization, and mortality [3-6]. One in seven patients undergoing chronic hemodialysis suffers a major bone fracture after a fall [7]. Additionally, fractures double the mortality risk in this population [5].

Unfortunately, falls are poorly understood in patients undergoing hemodialysis. Although some correlates have been identified, such as age, comorbidities, and number of medication burden [2,3], the ability to predict which patients will suffer falls remains limited. Improved risk prediction would allow for better patient care through prehabilitation and protective living arrangements.

We hypothesized that insights into falls derived from studies of the elderly might inform risk prediction in adults of all ages undergoing chronic hemodialysis; briefly stated, we hypothesized that frailty would be predictive of falls in this population. Frailty is a phenotype of physiological decline strongly associated with falls in the elderly [8,9]. We have identified frailty as important risk factor for mortality and hospitalization in patients undergoing dialysis of all ages; frailty was independently associated with a 2.60-fold higher risk of death and 1.43-fold higher number of hospitalizations [10]. To our knowledge, there are no studies assessing the association between frailty and falls in patients undergoing chronic hemodialysis. The primary goal of this study was to identify whether frailty was associated with falls independent of other risk factors for falls in patients undergoing hemodialysis of all ages.

## Methods

### Study design

This was a longitudinal study of 115 prevalent hemodialysis patients from a single dialysis center in Baltimore, Maryland, recruited between January 2009 and March 2010. Hemodialysis patients who were 18 years or older, spoke English, and agreed to participate were recruited into this study from a hospital-based outpatient dialysis unit. Frailty was measured at enrollment, and number of falls was self-reported at a follow-up visit in the 95 patients who survived at least 5 months. The follow-up visit was conducted at the dialysis center and was conducted on average within 6.7 months after enrollment. Demographics and comorbidities were obtained from medical record review at enrollment. The Johns Hopkins Institutional Review Board approved the study and all participants provided informed consent.

### Frailty

Frailty was measured as defined and validated by Fried et al. [8,11-20], as a phenotype based on 5 components: shrinking (self-report of unintentional weight loss of more than 10 lbs. in the past year based on dry weight); weakness (grip-strength below an established cutoff based on gender and BMI) [8]; exhaustion (self-report); low activity (Kcals/week below an established cutoff) [8]; and slowed walking speed (walking time of 15 feet below an established cutoff by gender and height) [8]. The 5 components of frailty were measured using previously described methods [8]. A score of 1 was given to those with the presence of each measured component. The aggregate frailty score was calculated as the sum of the component scores (range 0-5) and categorized as nonfrail (0-1), intermediately frail (2), and frail (3-5), as we previously established in ESRD patients [10].

### Statistical analysis

The association between frailty at enrollment and the number of falls reported during follow-up was estimated using Poisson regression. All adjusted models were adjusted for age, sex, and race. Additional models were also adjusted for comorbidity (as previously defined by Fried in the elderly and validated by our group in ESRD patients, namely 4 or more of the following conditions ascertained from medical records abstraction: peripheral vascular disease, rheumatoid arthritis, cancer, hypertension, COPD, diabetes, congestive heart failure, angina, and myocardial infarction) [8], disability in activities of daily living [21] (also previously described by Fried in the elderly and validated by our group in ESRD patients) [8], number of medications, education, and marital status at enrollment. Interactions between frailty and age were tested using a Wald test. Additionally, as sensitivity analyses we tested whether the results were similar when 1) we used negative binomial regression and 2) adjusted for other factors including anemia, arrhythmia, and cognitive function. All analyses were performed using STATA 12.0/MP for Linux (College Station, Texas).

## Results

### Study population

Among 95 study participants, the mean age was 60.5 years (SD=12.6), 43.6% were over 65, 46.3% were female, and 81.1% were African American; 25.3% of participants were nonfrail, 28.4% were intermediately frail, and 46.3% were frail. The median follow-up time was 6.7 months (range 5.0-13.3), which did not differ by frailty (6.7 months vs. 6.7 months, *P*=0.51) or falls (6.7 months vs. 6.7 months, *P*=0.98). The median time on dialysis was 3.7 years.

### Falls

**Figure 1:**
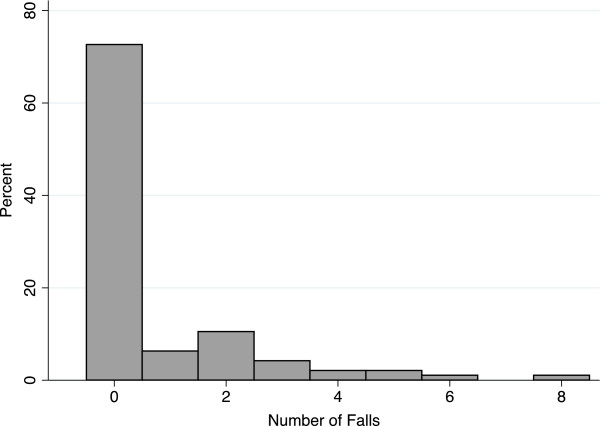
Distribution of number of falls in adults undergoing hemodialysis.

During follow-up, 28.3% of participants had one or more falls. The total number of falls was 70 and maximum number of falls a single participant experienced was 8 (Figure 1). Participants with one or more falls did not differ from those without falls based on comorbidity, number of medications, or education (Table 1). However, there were notable but not statistically significant differences in the number of years on chronic hemodialysis (no falls: 6.0 vs. falls: 4.0 years, *P*=0.17), percent of participants with prevalent disability (no falls: 17.4% vs. falls: 38.5%, *P*=0.054), and percent of participants who were married/cohabitating (no falls: 27.5% vs. falls: 46.2%, *P*=0.09). Age was not statistically associated with falls, with 25.9% of younger adults and 29.3% of older adults experiencing one or more falls (*P*=0.72).

**Table 1:** Characteristics of the study population, by falls during longitudinal follow-up

	**No falls (n=69)**	**Falls (n=26)**	***P *value**
Age (mean, SD)	59.9 [13.3]	62.2 [10.6]	0.44
Female (%)	44.9	50.0	0.82
Caucasian (%)	8.7	15.4	0.45
Enrollment body mass index (kg/m^2^) (mean, SD)	28.8 [8.6]	29.8 [7.9]	0.59
Pre-dialysis body mass index (kg/m^2^) (mean, SD)	29.9 [10.2]	32.0 [9.1]	0.37
Smoking history (%)	18.8	19.2	0.99
Years on hemodialysis (mean, SD)	6.0 [7.0]	4.0 [3.3]	0.17
Married/cohabitating (%)	27.5	46.2	0.09
Education (% high school or higher)	82.6	88.5	0.75
Comorbidities (%)			
Peripheral vascular disease	31.9	23.1	0.46
Rheumatoid arthritis	7.3	11.5	0.68
History of cancer	17.4	26.9	0.38
Hypertension	91.3	88.5	0.70
Chronic obstructive pulmonary disease	15.9	26.9	0.25
Diabetes	66.7	76.9	0.46
Congestive heart failure	29.1	42.3	0.82
Angina	5.8	7.7	0.66
Myocardial infarction	17.4	15.4	0.99
Number of medications (mean, SD)	8.7 [3.4]	8.1 [3.1]	0.42
Comorbidity (%)^1^	30.4	34.6	0.81
Disability (%)^2^	17.4	38.5	0.05

^1^Comorbidity = 4 or more of the following conditions: peripheral vascular disease, rheumatoid arthritis, cancer, hypertension, COPD, diabetes, congestive heart failure, angina, and myocardial infarction.

^2^Disability = Needing assistance on 3 or more of the following activities: feeding, dressing, ambulation, grooming, using a toilet, and bathing.

### Frailty and falls

Frailty predicted a 3.55-fold (95% CI: 1.68-7.46, *P*=0.001) higher number of falls in the unadjusted model and predicted a 3.89-fold (95% CI: 1.78-8.49, *P*=0.001) higher number of falls after adjusting for age, sex, and race (Table 2). In a separate model that was additionally adjusted for comorbidity, disability, number of medications, education, and marital status, frailty predicted a 3.09-fold (95% CI: 1.38-6.90, *P*=0.006) higher number of falls. In all models, intermediate frailty was not itself associated with an increased number of falls, but there was a statistically significant trend in the risk of falls from nonfrail to intermediately frail to frail (all *P*≤0.001). The association of frailty and number of falls did not differ for younger and older adults (*P*=0.57 for interaction between age and frailty).

**Table 2:** Factors associated with falls in adults undergoing hemodialysis

	**Unadjusted**	**Parsimonious model**	**Full model**
Frailty status			
Nonfrail	Reference	Reference	Reference
Intermediately frail	1.11 (0.44, 2.82)	1.35 (0.52, 3.49)	1.19 (0.44, 3.24)
Frail	3.55 (1.68, 7.46)**	3.89 (1.78, 8.49)**	3.09 (1.38, 6.90)**
Age (in 10 years)	-	0.83 (0.68, 1.01)	0.90 (0.69, 1.17)
Female	-	1.68 (1.01, 2.79)*	1.84 (1.06, 3.19)*
Caucasian	-	0.98 (0.46, 2.12)	0.84 (0.37, 1.91)
Comorbidity^1^	-	-	0.88 (0.47, 1.65)
Disability^2^	-	-	1.71 (0.97, 3.00)
Medication use^3^	-	-	0.91 (0.85, 0.98)*
High school education or higher	-	-	3.63 (1.29, 10.24)*
Marital status			
Single	-	-	Reference
Married/cohabitating	-	-	1.71 (0.86, 3.39)
Separated/divorced	-	-	1.13 (0.49, 2.61)
Widowed	-	-	1.10 (0.43, 2.81)

^1^Comorbidity = 4 or more of the following conditions: peripheral vascular disease, rheumatoid arthritis, cancer, hypertension, COPD, diabetes, congestive heart failure, angina, and myocardial infarction.

^2^Disability = Needing assistance on 3 or more of the following activities: feeding, dressing, ambulation, grooming, using a toilet, and bathing.

^3^Medication use reflects the association of a 1 medication increase and number of falls.

**P <0.01.

*P <0.05.

### Sensitivity analyses

The association of frailty and falls was robust to the statistical methods used. Additionally, the adjustment for anemia (frailty: 2.64, 95% CI: 1.17-5.98), arrhythmias (frailty: 3.04, 95% CI: 1.36-6.80), and low cognitive function (frailty: 3.02, 95% CI: 1.35-6.78) did not change the inferences on the association of frailty and number of falls.

## Discussion

In this single-center study, 28.3% of prevalent hemodialysis patients of all ages had a fall. Frailty was a strong independent predictor of falls in this population. Frail patients undergoing chronic hemodialysis experienced 3-times as many falls as their non-frail counterparts. Frailty was a strong independent predictor of falls in patients undergoing hemodialysis, regardless of age.

Elderly hemodialysis patients have been found to be at increased risk of falls [22], and thus, most research has focused on falls in this vulnerable patient population [1,3,6]. Our incidence estimates of falls in patients undergoing hemodialysis are similar to previous studies [1], but we also present data that these estimates are similar even in younger hemodialysis patients. In addition to previously reported predictors of falls (age, comorbidity, and medication use) we have introduced frailty as a novel predictor of falls in both younger and older patients undergoing chronic hemodialysis. In fact, in our study frailty was one of the strongest predictors of falls in our adjusted model. This study not only confirms the association between frailty and falls observed in elderly adults [8,9,23] but extends the findings to patients undergoing hemodialysis of all ages. In older adults, frailty has been hypothesized to be a unique disorder that is independent of chronic conditions and disability. Our work confirms that frailty is an independent phenotype and predictive of falls, regardless of comorbidities and disability for patients undergoing hemodialysis.

Frailty, as defined by Fried et al., is a physiologic precursor and an etiologic factor in the onset of disability [8]. Frailty is thought to lead to disability and subsequent falls due to weakness and low endurance. Although frailty is a distinct phenotype from disability, frailty begins affecting mobility before clinically important outcomes like falls. Thus, the onset of frailty may be the optimal time to initiate interventions to prevent disabilities in mobility and prevent falls.

Strengths of this study were the direct measurement of a validated, objective frailty instrument, and granular ascertainment of comorbidities using medical records abstraction. Additionally, frailty was ascertained prior to the report of falls, so its predictive value could be assessed. The main limitation is the single-center study design, so direct inferences must be interpreted in the context of the demographics of our study population. While this study was conducted in a hospital-based outpatient dialysis unit in Baltimore, the findings are likely generalizable to patients undergoing hemodialysis in a community setting across the US. Additionally, the sample size of the cohort was small, which will not bias the observed associations, but did lead to low power to detect subgroup differences. Although follow-up was limited, we were interested in ascertaining the number of falls within 6 months from the measurement of frailty, which does not require longer follow-up. We had limited ability to adjust for a number of factors that may be associated with falls (regardless of the association with frailty); however, the strength of the association between frailty and falls was similar when adjusted for anemia, arrhythmia, and cognitive function. Finally, survivor bias limits inferences from our finding that frailty was not associated with time on hemodialysis.

## Conclusions

Frailty was identified as a novel risk factor for falls in adults of all ages undergoing hemodialysis. Additionally, the results suggest that among older and younger patients undergoing hemodialysis, frailty increases the short-term risk of experiencing a fall. Our results may aid in identifying frail hemodialysis patients who could be targeted for multidimensional fall prevention strategies.

## Abbreviations

ESRD: End stage renal disease; COPD: Chronic obstructive pulmonary disease.

## Competing interests

The authors declare that they have no competing interest.

## Authors’ contributions

All authors 1) have made substantial contributions to conception and design, or acquisition of data, or analysis and interpretation of data; 2) have been involved in drafting the manuscript or revising it critically for important intellectual content; and 3) have given final approval of the version to be published.

## Pre-publication history

The pre-publication history for this paper can be accessed here:

http://www.biomedcentral.com/1471-2369/14/224/prepub

## References

[B1] Cook WL Jassal SV Prevalence of falls among seniors maintained on hemodialysis Int Urol Nephrol 2005 37 3 649 652 10.1007/s11255-005-0396-9 16307356

[B2] Desmet C Beguin C Swine C Jadoul M Falls in hemodialysis patients: prospective study of incidence, risk factors, and complications Am J Kidney Dis 2005 45 1 148 153 10.1053/j.ajkd.2004.09.027 15696454

[B3] Cook WL Tomlinson G Donaldson M Markowitz SN Naglie G Sobolev B Jassal SV Falls and fall-related injuries in older dialysis patients Clin J Am Soc Nephrol 2006 1 6 1197 1204 10.2215/CJN.01650506 17699348

[B4] Leinau L Perazella MA Hip fractures in end-stage renal disease patients: incidence, risk factors, and prevention Semin Dial 2006 19 1 75 79 10.1111/j.1525-139X.2006.00122a.x 16423185

[B5] Mittalhenkle A Gillen DL Stehman-Breen CO Increased risk of mortality associated with hip fracture in the dialysis population Am J Kidney Dis 2004 44 4 672 679 15384018

[B6] Li M Tomlinson G Naglie G Cook WL Jassal SV Geriatric comorbidities, such as falls, confer an independent mortality risk to elderly dialysis patients Nephrol Dial Transplant 2008 23 4 1396 1400 18057068 10.1093/ndt/gfm778

[B7] Kohlmeier M Saupe J Schaefer K Asmus G Bone fracture history and prospective bone fracture risk of hemodialysis patients are related to apolipoprotein E genotype Calcif Tissue Int 1998 62 3 278 281 10.1007/s002239900430 9501964

[B8] Fried LP Tangen CM Walston J Newman AB Hirsch C Gottdiener J Seeman T Tracy R Kop WJ Burke G Frailty in older adults: evidence for a phenotype J Gerontol A Biol Sci Med Sci 2001 56 3 M146 M156 10.1093/gerona/56.3.M146 11253156

[B9] Ensrud KE Ewing SK Taylor BC Fink HA Stone KL Cauley JA Tracy JK Hochberg MC Rodondi N Cawthon PM Frailty and risk of falls, fracture, and mortality in older women: the study of osteoporotic fractures J Gerontol A Biol Sci Med Sci 2007 62 7 744 751 10.1093/gerona/62.7.744 17634322

[B10] McAdams-DeMarco MA Law A Salter ML Boyarsky B Gimenez L Jaar BG Walston JD Segev DL Frailty as a novel predictor of mortality and hospitalization in individuals of all ages undergoing hemodialysis J Am Geriatr Soc 2013 61 6 896 901 10.1111/jgs.12266 23711111 PMC3938084

[B11] Bandeen-Roche K Xue QL Ferrucci L Walston J Guralnik JM Chaves P Zeger SL Fried LP Phenotype of frailty: characterization in the women's health and aging studies J Gerontol A Biol Sci Med Sci 2006 61 3 262 266 10.1093/gerona/61.3.262 16567375

[B12] Barzilay JI Blaum C Moore T Xue QL Hirsch CH Walston JD Fried LP Insulin resistance and inflammation as precursors of frailty: the Cardiovascular Health Study Arch Intern Med 2007 167 7 635 641 10.1001/archinte.167.7.635 17420420

[B13] Cappola AR Xue QL Fried LP Multiple hormonal deficiencies in anabolic hormones are found in frail older women: the Women's Health and Aging studies J Gerontol A Biol Sci Med Sci 2009 64 2 243 248 19182229 10.1093/gerona/gln026 PMC2655016

[B14] Leng SX Hung W Cappola AR Yu Q Xue QL Fried LP White blood cell counts, insulin-like growth factor-1 levels, and frailty in community-dwelling older women J Gerontol A Biol Sci Med Sci 2009 64 4 499 502 19251912 10.1093/gerona/gln047 PMC2657176

[B15] Leng SX Xue QL Tian J Walston JD Fried LP Inflammation and frailty in older women J Am Geriatr Soc 2007 55 6 864 871 10.1111/j.1532-5415.2007.01186.x 17537086

[B16] Newman AB Gottdiener JS McBurnie MA Hirsch CH Kop WJ Tracy R Walston JD Fried LP Associations of subclinical cardiovascular disease with frailty J Gerontol A Biol Sci Med Sci 2001 56 3 M158 M166 10.1093/gerona/56.3.M158 11253157

[B17] Walston J McBurnie MA Newman A Tracy RP Kop WJ Hirsch CH Gottdiener J Fried LP Frailty and activation of the inflammation and coagulation systems with and without clinical comorbidities: results from the Cardiovascular Health Study Arch Intern Med 2002 162 20 2333 2341 10.1001/archinte.162.20.2333 12418947

[B18] Xue QL Bandeen-Roche K Varadhan R Zhou J Fried LP Initial manifestations of frailty criteria and the development of frailty phenotype in the Women's Health and Aging Study II J Gerontol A Biol Sci Med Sci 2008 63 9 984 990 10.1093/gerona/63.9.984 18840805

[B19] Chang SS Weiss CO Xue QL Fried LP Association between inflammatory-related disease burden and frailty: results from the Women's Health and Aging Studies (WHAS) I and II Arch Gerontol Geriatr 2012 54 1 9 15 10.1016/j.archger.2011.05.020 21763008 PMC3197795

[B20] Chang SS Weiss CO Xue QL Fried LP Patterns of comorbid inflammatory diseases in frail older women: the Women's Health and Aging Studies I and II J Gerontol A Biol Sci Med Sci 2010 65 4 407 413 19933749 10.1093/gerona/glp181 PMC3004772

[B21] Katz S Akpom CA A measure of primary sociobiological functions Int J Health Serv: Plann, Adm, Eval 1976 6 3 493 508 10.2190/UURL-2RYU-WRYD-EY3K 133997

[B22] Roberts R Jeffrey C Carlisle G Brierley E Prospective investigation of the incidence of falls, dizziness and syncope in haemodialysis patients Int Urol Nephrol 2007 39 1 275 279 10.1007/s11255-006-9088-3 17318349

[B23] Shim EY Ma SH Hong SH Lee YS Paik WY Seo DS Yoo EY Kim MY Yoon JL Correlation between frailty level and adverse health-related outcomes of community-dwelling elderly, One year retrospective study Korean J Fam Med 2011 32 4 249 256 10.4082/kjfm.2011.32.4.249 22745861 PMC3383131

